# Spinal Cord T-Cell Infiltration in the Rat Spared Nerve Injury Model: A Time Course Study

**DOI:** 10.3390/ijms17030352

**Published:** 2016-03-09

**Authors:** Christophe Gattlen, Christine B. Clarke, Nicolas Piller, Guylène Kirschmann, Marie Pertin, Isabelle Decosterd, Romain-Daniel Gosselin, Marc R. Suter

**Affiliations:** 1 Pain Center, Department of Anesthesiology, Lausanne University Hospital (CHUV) and University of Lausanne, 1011 Lausanne, Switzerland; Nicolas.Piller@chuv.ch (N.P.); Guylene.Kirschmann@unil.ch (G.K.); Marie.Pertin@unil.ch (M.P.); Isabelle.Decosterd@chuv.ch (I.D.); rdg@biotelligences.com (R.-D.G.); 2 Department of Fundamental Neurosciences, University of Lausanne, 1005 Lausanne, Switzerland

**Keywords:** peripheral nerve injury, SNI (spared nerve injury), SCI (spinal cord injury), microglia, lymphocytes, neuropathic pain

## Abstract

The immune system is involved in the development of neuropathic pain. In particular, the infiltration of T-lymphocytes into the spinal cord following peripheral nerve injury has been described as a contributor to sensory hypersensitivity. We used the spared nerve injury (SNI) model of neuropathic pain in Sprague Dawley adult male rats to assess proliferation, and/or protein/gene expression levels for microglia (Iba1), T-lymphocytes (CD2) and cytotoxic T-lymphocytes (CD8). In the dorsal horn ipsilateral to SNI, Iba1 and BrdU stainings revealed microglial reactivity and proliferation, respectively, with different durations. Iba1 expression peaked at D4 and D7 at the mRNA and protein level, respectively, and was long-lasting. Proliferation occurred almost exclusively in Iba1 positive cells and peaked at D2. Gene expression observation by RT-qPCR array suggested that T-lymphocytes attracting chemokines were upregulated after SNI in rat spinal cord but only a few CD2/CD8 positive cells were found. A pronounced infiltration of CD2/CD8 positive T-cells was seen in the spinal cord injury (SCI) model used as a positive control for lymphocyte infiltration. Under these experimental conditions, we show early and long-lasting microglia reactivity in the spinal cord after SNI, but no lymphocyte infiltration was found.

## 1. Introduction

Neuropathic pain is a type of chronic pain, arising as a consequence of a lesion or disease of the somatosensory nervous system. It affects up to 10% of the global population [1] and, therefore, represents a major challenge for the medical community. Although the lesion occurs in neurons, glial and immune cells (such as astrocytes, microglia, infiltrating macrophages or lymphocytes) are implicated in chronic pain in both the periphery and in the central nervous system (CNS) [2,3,4,5].

When a peripheral nerve is injured, an immune reaction occurs, with an initial activation of neuroglial cells in the dorsal horn of the spinal cord, ipsilateral to the injury. In particular, microglia (the CNS macrophages) proliferate and change their morphology and gene expression (such as an up-regulation of Iba1) [3,4,5]. Following peripheral nerve injury, microglia activation is often described as early and transient participating in the development of pain-related behavior, whereas the response of other immune-mediating cells, such as astrocytes, begins later and contributes to the maintenance of the chronic pain [5]. Using chimera mice with a partial sciatic nerve ligation (PSNL), Zhang *et al.* showed that a number of cells expressing microglia markers were of hematogenous monocytic origin, infiltrating the spinal cord before proliferating there [6]. Leukocyte trafficking through the blood–brain barrier has also been reported after L5 spinal nerve transection in rats [7]. Many studies have been performed using different neuropathic pain models, including chronic constriction injury (CCI) [8], L5 spinal nerve transection [9], and spared nerve injury (SNI) [10] in the investigation of lymphocyte infiltration, but this phenomenon was not observed in all studies and therefore remains somewhat controversial [11,12]. The exact contribution of infiltrating lymphocytes in neuropathic pain needs further study.

Here, we aim to characterize some specific features of neuroimmune reactivity in the spinal cord following peripheral nerve injury in rats. For this study, we used the SNI model of neuropathic pain in Sprague Dawley adult male rats to assess proliferation, and/or protein/gene expression levels for microglia (Iba1), T-lymphocytes (CD2) and cytotoxic T-lymphocytes (CD8). We observed the reaction of the immune system over a prolonged time course performing an array study of inflammatory markers as well as immunostainings of lymphocyte infiltration into the spinal cord.

## 2. Results

### 2.1. SNI Upregulates Genes Related to Microglial Reactivity

**Figure 1:**
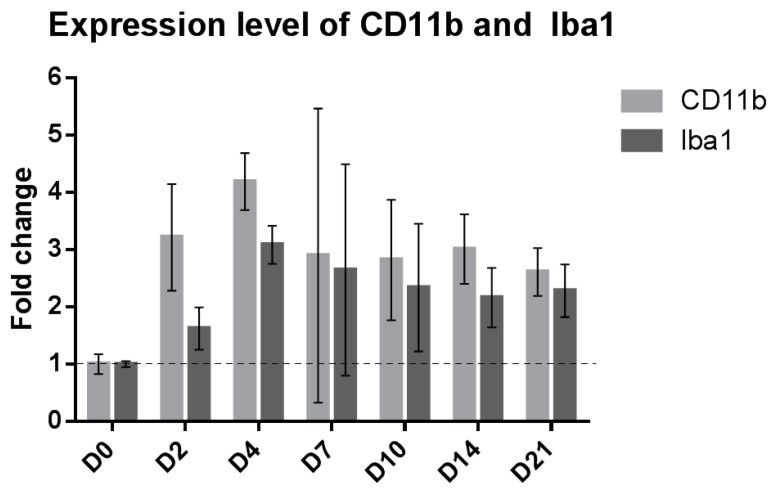
Upregulation of microglial markers after SNI. Bar graph showing the mRNA fold change in rat ipsilateral dorsal horns of the microglial markers CD11b (light gray) and Iba1 (dark gray) compared to naive animals. Values are expressed as mean ± 95% CI. *N* = 3 for each time point.

Microglial reactivity was assessed using a gene expression time course of the microglial markers CD11b and Iba1 in the lumbar section of adult male Sprague Dawley rats 2, 4, 7, 10, 14 and 21 days after SNI compared to naive animals. We observed an upregulation of CD11b at day 2, 4, 10, 14 and 21, and an upregulation of Iba1 at day 2, 4, 10, 14 and 21 (non-overlap of 95% CI method compared to D0 [13], Figure 1, no sham group was used for each time point to lower the number of animals needed).

**Figure 2:**
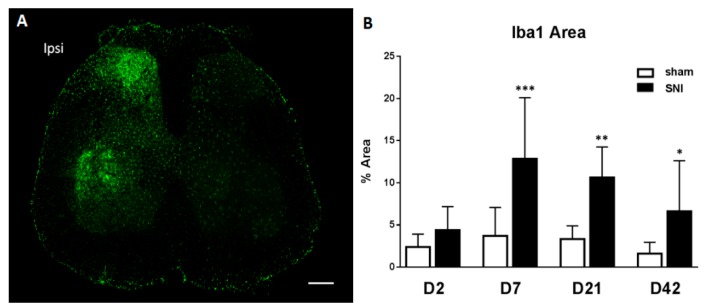
Neuroinflammation in the spinal cord after SNI. (**A**) Immunofluorescence of a lumbar section seven days after injury showing the microglial activation marker Iba1 in green (**B**) Time course of the percentage of Iba1 positive area by immunostaining in the ipsilateral dorsal horn. *N* = 4/group. Values are expressed as mean ± 95% CI, 2-way ANOVA with Sidak correction, sham *vs*. SNI. * *p* < 0.05, ** *p* < 0.01, *** *p* < 0.001. Scale bar represents 200 µm.

We also assessed the change over time of Iba1 at the protein level using immunofluorescence on the ipsilateral dorsal horn of the spinal cord: the time course of Iba1 protein expression follows that of mRNA but with a few days delay, peaking at day 7 (Figure 2). At both the mRNA and protein level we observe a prolonged increase in microglial reactivity.

### 2.2. SNI Induces Microglial Activation and Proliferation

**Figure 3:**
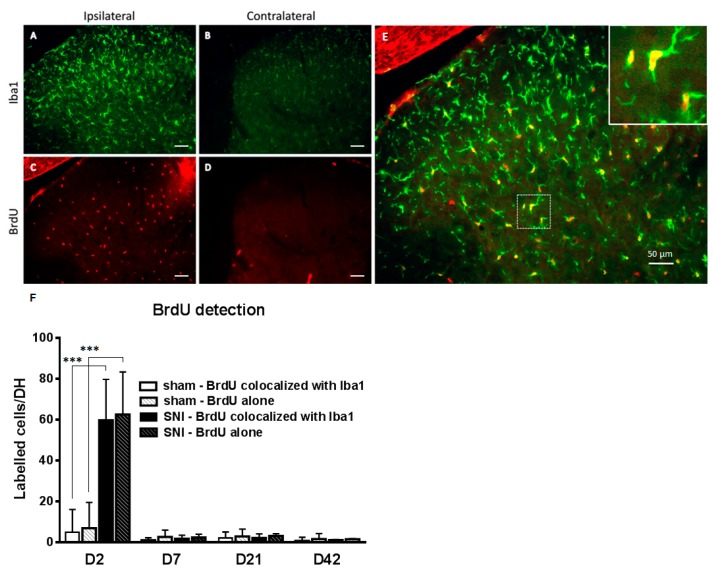
Colocalization of the proliferation marker BrdU with Iba1 in the ipsilateral dorsal horn two days after SNI. (**A**–**D**) Iba1 (green) and BrdU (red) in the ipsilateral (**A**,**C**) and contralateral (**B**,**D**) dorsal horn two days after SNI surgery. There is a marked microglial reaction on the ipsilateral side (**E**) Merge of Iba1 and BrdU ipsilateral showing the colocalization of the two markers indicating microglial proliferation following peripheral nerve injury. Insert: magnification (**F**) Time course of BrdU positive cells in dorsal horn, colocalized or not with Iba1. *N* = 4/group. Values are expressed as mean ± 95% CI, 2-way ANOVA with Sidak correction, *** *p* < 0.001. DH, dorsal horn. Scale bar represents 50 µm.

We also investigated microglial proliferation as an indicator of microglial reactivity. Using immunofluorescence, we performed an expression time course of the proliferation marker BrdU (bromodeoxyuridine) on rat spinal cord lumbar sections combined with the cellular marker Iba1 (Figure 3). We observed a peak of the BrdU signal at D2. BrdU signal almost fully (95.5%) colocalized with Iba1 (Figure 3F).

The time course of proliferation, with a sharp increase only observed at day 2, is in stark contrast to the prolonged increase of the markers Iba1 and CD11b. This highlights the necessity to specify how microglial reactivity is defined. Using only proliferation as a marker of reactivity could lead to the misleading idea that microglial activation is restricted to early time points after peripheral nerve injury.

### 2.3. Inflammatory Genes Are Regulated after Peripheral Nerve Injury

**Figure 4:**
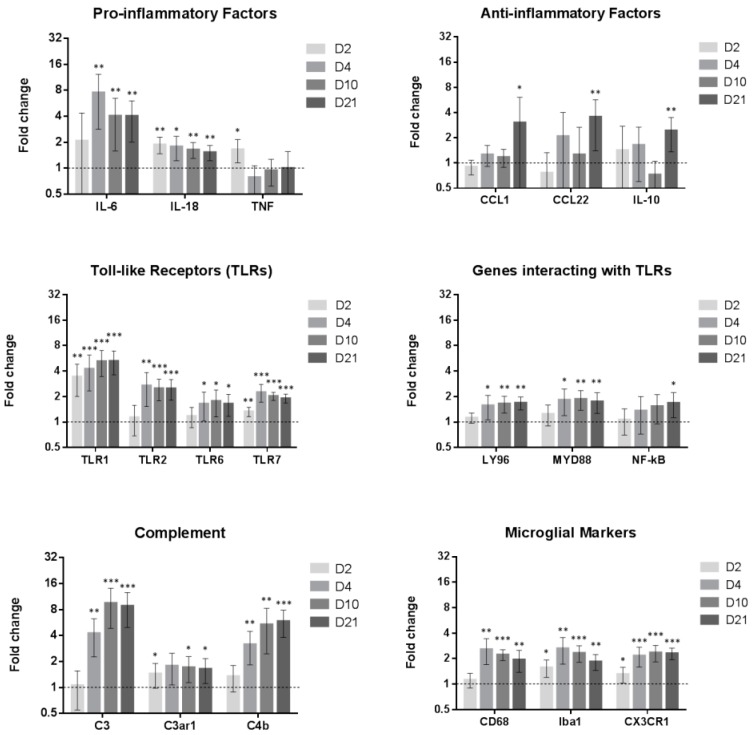
Gene regulation in the spinal cord following SNI. RT-qPCR array with fold change of gene expression in adult male rat ipsilateral dorsal horns at D2, D4, D10 and D21 after SNI compared to naive animals. Values are expressed as mean ± 95% CI. Dotted line at one fold regulation (naive animals). * *p* < 0.05, ** *p* < 0.01, *** *p* < 0.001. Student’s *t*-test: Naive (not shown) vs SNI. *N* = 6 for each time point (D2, D4, D10 and D21).

Based on the Iba1 expression time course (Figure 1), we selected four time points (2, 4, 10 and 21 days) at which to assess the mRNA expression changes of 96 genes of interest in the lumbar spinal cord of adult male Sprague Dawley rats in SNI compared to naive animals (Figure 4). Sham animals were only tested for 2 and 4 day and are shown in the supplemental data. These are the time points with the highest chances to demonstrate modification caused by inflammation. Differences for TLR1 and Iba1 in sham D2 and for CCL12 in sham D4 were significant. Long term changes might have been missed by not performing sham D10 and D21. Exact p-values are available in Table S1.

Genes were associated in clusters:

Prototypical pro-inflammatory factors such as TNF-α or IL-6 [14] were upregulated at early time points. At later time points, such as day 21, we noticed the upregulation of more “anti-inflammatory factors” such as IL-10, CCL1 or CCL22 which could play a role in the resolution of inflammation [15,16].

Most of the toll-like receptors (TLRs) are upregulated during the whole time course (see in Supplementary Figure S2).Genes involved in the complement system are also upregulated following nerve injury. We observed, in this study, changes in C3 and C4b expression, confirming previous studies [17]. The expression of the microglial markers Iba1, CD68 and CX3CR1, used to indicate microglial reactivity, were also assessed. Their mRNA was upregulated over the full time course (Supplementary Figures S2 and S3. The full array data are available at figshare [18]).

**Figure 5:**
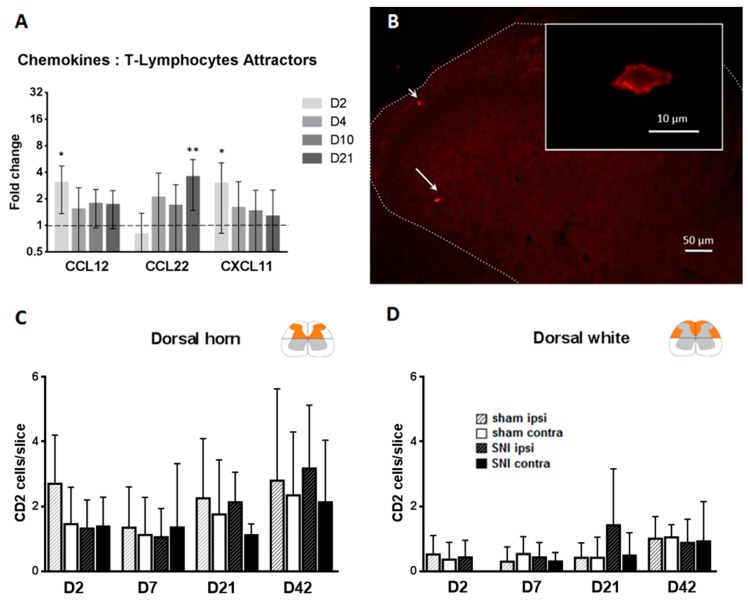
CD2-positive T-cell infiltration in the lumbar spinal cord. (**A**) Expression of genes of T-cells attractors in the spinal cord of rats following SNI from the RT-qPCR array showing the upregulation of gene expression of T-lymphocytes attractors. * *p* < 0.05, ** *p* < 0.01. Student’s *t*-test: Naive (not shown) *vs.* SNI. *N* = 6 for each time point (D2, D4, D10 and D21); (**B**) Immunofluorescence of the ipsilateral dorsal horn showing CD2+ cells (pointed by arrows) in the spinal cord seven days after SNI; Insert: Magnification of the cell pointed by the longest arrow (**C**,**D**) Bar histograms showing the number of CD^2+^ cells detected in sham or SNI at days 2, 4, 10 and 21 in the ipsilateral or contralateral side to SNI. Two regions of spinal cords were analyzed: **C**, the dorsal horn and **D**, the dorsal white matter. *N* = 4/groups. Values are expressed as mean ± 95% CI. No significant difference, 2-way ANOVA with Sidak correction.

As Lymphocyte/T-lymphocyte attractors [19], such as CCL12 [20], CXCL11 [21] or CD74 [22], were upregulated (Figure 5A), we wanted to further investigate if there was indeed an infiltration of lymphocytes through the blood–spinal cord barrier. Counting regions have been defined in Supplementary Figure S1. Injured nerve regions have been verified with isolectin B4 (see Supplementary Figure S6).

### 2.4. T-Cells Do Not Infiltrate the Spinal Cord after SNI

To detect T-cell infiltration after SNI, we counted either CD2, a general marker for T-lymphocytes or CD8 a marker of cytotoxic T-lymphocytes in different parts of the dorsal cord using immunofluorescence (Figure 5B–D and Supplementary Figure S5). There were only very few CD2-positive T-cells in our slices. We did not find a significant difference between the ipsilateral and the contralateral side of the dorsal horn at any time point. The only significant difference between sham and SNI was found in the dorsal white matter, with an absolute number of 1.5 cells per slice. Under our experimental conditions, there was no T-cell infiltration into the dorsal horn after SNI.

### 2.5. T-Cells Infiltrate the Spinal Cord after SCI

**Figure 6:**
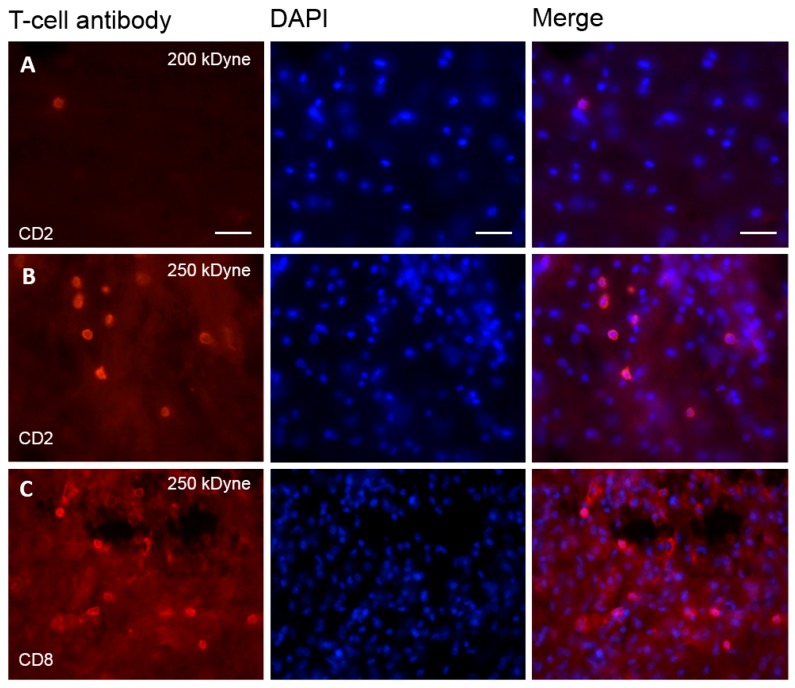
Infiltration of the spinal cord by T-cells after SCI. (**A**,**B**) Immunofluorescence of CD2^+^ T-cells seven days after a SCI induced with a strength of 200 and 250 kdyn, respectively (machine range: 30–300 kdyn); (**C**) Immunofluorescence of CD8^+^ T-cells after a SCI induced with a strength of 250 kdyn. We observe a clear T-cell infiltration with both CD2 and CD8 antibodies at 250 kdyn SCI. Scale bar represents 50 µm.

To confirm whether we were actually able to observe T-cell infiltration, we applied the same methodology to the spinal cord injury model (SCI), which consists of a direct mechanical injury to the spinal cord. We could indeed observe CD2 and CD8-positive cells following a 200 kdyn force injury and a high number of these cells following the 250 kdyn injury (Figure 6A–C).

## 3. Discussion

We here show novel findings in inflammatory changes in the spinal cord after peripheral nerve injury. We first show a prolonged increase in microglial markers at the mRNA (Iba1, CD11b) and protein level (Iba1). However, proliferation of microglia is only increased at day 2. We observed a switch from pro-inflammatory factors upregulated early, to anti-inflammatory factors later. Finally, we did not detect lymphocyte infiltration in the spinal cord.

Different classifications of microglia reactivity were attempted, mostly paralleling the classifications used for circulating macrophages. They vary from simply dichotomizing between a classical M1 and alternative M2, to much broader description of a continuum of phenotypes using immunological markers as well as functional aspects (phagocytosis, antigen presentation, cytokine secretion) [16,23,24]. Iba1 and CD11b are cell markers not usually used to discriminate between any subtypes. CD68 is a lysosomal marker indicative of microglial phagocytic activity [25]. CX3CR1 is the receptor for fractalkine (CX3CL1) solely expressed in microglia in the central nervous system and participating in the pathophysiology of pain following nerve injury [26]. These microglial markers show a longstanding increase over at least 21 or 42 days. The pro-inflammatory cytokines IL-1β, TNF-α and IL-6 have been shown to increase excitability in lamina II neurons of the spinal cord [27]. These cytokines were also shown to modulate the phenotype of microglia. Interestingly, IL-10 and IL-6 were shown to modulate towards alternative phenotypes. This highlights the ambiguous aspect of IL-6, which is upregulated until day 21 in our array and might have a dual role in neurons and microglia [28]. For the more classical anti-inflammatory IL-10, which has recently been shown to be responsible for decreased neuropathic pain in rat pups compared to adults [29] and used successfully to treat neuropathic pain [30], the increase in the late phase could correspond to a switch from M1 to alternative phenotype. The cell-type responsible for the regulation is unknown in whole tissue samples, which is a limitation of the array study part of our work.

Nociceptive information is relayed by many mechanisms including pattern recognition systems, such as Toll-like receptors (TLRs) [31]. TLRs play an important role in microglial activation following the detection of danger or damage-associated molecules. These can be exogenous (such as bacterial lipopolysaccharides (LPS), viral glycoproteins, bacterial peptidoglycan, or parasitic/viral RNAs) or endogenous (such as proteins like fibronectin, polysaccharides like heparan sulphate, nucleic acids and phospholipids) released upon tissue damage or cell death [32]. TLRs allow, via a downstream cascade, the release of pro-inflammatory factors including IL-1β, IL-6, IL-18, and TNF-α [33]. Many of these factors were upregulated after SNI in our study (Figure 4). TLR2 is an important microglial activator, using both ERK and NF-κB pathways [34], TLR4 and TLR7 also contribute to the process by allowing the release of pro-inflammatory factors such as IL-1β, TNF-α through the NF-κB pathway [33,35].

The complement cascade has been shown to be activated after peripheral nerve injury and to play a role in hypersensitivity through the release of the anaphylatoxin peptide (C5a) by the terminal membrane attack complex (MAC) complement, itself induced by C3 activity [17]. We observe an increase in complement factors which confirms this literature and supports our array data.

We only used sham animals at day 2 and 4, to follow the 3Rs recommendations. We thought that inflammatory changes in sham animals, if they occur, would be present at the early time points (shown in Supplementary Figure S3). We acknowledge the limitation that later changes in sham animals could have been missed.

Lymphocyte infiltration into the spinal cord after a peripheral injury is still debated. We do not find relevant CD2, neither CD8 positive lymphocytes infiltration following SNI. The only significant difference between sham and SNI is seen at day 21 in the dorsal part of the white matter for CD2 positive cells, with less than 1.5 cells/slice. Our study differs from others, where different model/species were used, such as the sciatic nerve transection or the CCI in rats [8] or spinal nerve transection in mice [10], and where immune cells infiltration through the blood spinal cord barrier [8,10] was indeed detected. However, no T-cells were observed after PSNL in mice (11) or CCI in rats (12) in line with our results. Costigan *et al.* used the same model, time point, rat strain and gender, and antibodies as we did [10], and demonstrated the infiltration of CD2 positive cells into the spinal cord. Our detection technique differed slightly from that used in the Costigan study. However, we demonstrated our ability to detect both CD2 and CD8 positive cells using the SCI model, minimizing the risk of a false negative, due to a different lot of antibody. We sampled spinal cord sections regularly across the spinal cord where IB4 staining was lost and we therefore reduced the risk of missing the part of interest [3]. Recently, gender has been surprisingly reported as a major driver for different immune reactions following nerve injury [36]. In that study, female mice were using adaptive immunity, likely T-lymphocytes, and not male mice. They did not quantify infiltration but show higher levels of CD3e, CD4 and CD8a mRNA in female mice than in male mice. Conceptually, this paper underlines the fact that different pathways are involved in pain development which can replace one another as shown by the different paradigms they test when changing the hormonal status of their animals. We can therefore hypothesize that other factors, not yet identified, could explain the differential results compared to the data of Costigan *et al.* Housing conditions influence microglia phenotype through the germ prevalence or [37,38]. Differences in handling, stress or food could also be contributing factors.

To conclude, we identified an early expression of pro-inflammatory factors and a later expression of anti-inflammatory markers in the spinal cord after SNI. Finally, despite showing an increase in lymphocyte-attracting chemokines, we did not observe an increase in CD2 and CD8 positive cells in the spinal cord following SNI. We have shown that there is a longstanding upregulation of microglial reactivity markers (CD11b and Iba1) up to 42 days, whereas proliferation is only observed shortly. This highlights that when microglia reactivity is mentioned, it has to be defined very specifically as it varies over time. Further research should focus on specific changes in microglia, and try to understand not only the early pro-inflammatory reaction, but also the active switch to resolution of inflammation.

## 4. Materials and Methods

### 4.1. Animal Surgery

Adult male Sprague Dawley rats (250–350 g) were anesthetized by isoflurane. We used the spared nerve injury (SNI) model of neuropathic pain [39]: After an incision through the skin and muscle and the visualization of the sciatic trifurcation, the sural nerve was left intact and the two other sciatic nerve branches (the common peroneal and the tibial nerve) were ligated, cut distally with a few millimeters removed. The wounds were then closed separately for muscle and skin and rats were placed back in their cage. The same procedure was followed for sham surgery, but the nerves were not cut; instead, a silk was laid next to the trifurcation of the sciatic nerve. Spinal cord injury (SCI) was performed under aseptic conditions and general anesthesia: a partial laminectomy was made at the L4–L5 lumbar level of spinal cord and a 250 or 200 kdyn (1 dyn = 10 μN) contusion injury was applied using a force-controlled spinal cord impactor (IH-0400 Impactor, Precision Systems and Instrumentation (Nottinghill, VA, USA) [40], using a holding device [41]. All procedures were approved by the committee on animal experimentation for the canton of Vaud, Switzerland, in accordance with Swiss federal law on animal welfare and guidelines of the international association for the study of pain (IASP) [42].

### 4.2. Immunohistochemistry

We injected intraperitoneally (i.p.) 25mg of bromodeoxyuridine (BrdU) in phosphate buffered saline (PBS) + 0.007 N NaOH 2 h before sacrifice. Rats were lethally anesthetized by a 50 mg i.p. injection of pentobarbital and perfused with ice cold PBS for 1 min followed by 4% paraformaldehyde (PFA) PBS for 4 min.

The ipsilateral sciatic nerve was dissected and exposed up to the L4–L5–L6 spinal nerves, then the spinal cord was exposed and the L4-L5 lumbar section was collected and post-fixed in 4% PFA at 4 °C overnight. Samples were then transferred into 20% sucrose overnight for cryoprotection and then rapidly frozen. Using a cryostat, 30 μm slices were cut and stored in 30% glycol and 30% glycerol PBS. For immunofluorescence, slices were incubated for 30 min in blocking solution: 10% goat serum (NGS), 0.05% triton in 0.05% azide PBS, and overnight with the primary antibody (Table 1).

After washing in PBS, slices were incubated in the blocking solution for one hour at room temperature with secondary antibody (Table 2). Diamidinophenylindole (DAPI) was applied on the slices for nuclear labelling before the final PBS wash. Lumbar spinal cord sections were then mounted on slides with mowiol mounting medium and stored at 4 °C.

To verify that the part of the spinal cord corresponds to the topographic region of lumbar dorsal horn occupied by the central terminals of afferent injured nerves, a staining with isolectin (Griffonia simplicifolia) B4 FITC conjugated has been performed [3]. One of each 5 slices has been stained. After blocking the slice for half an hour, the IB4 was incubated for 2 h, the slices were then washed in PBS and mounted in mowiol.

Thymi and neuromas were also collected for positive controls for antibody testing (See Supplementary Figure S4).

For cell counting and signal quantification, six sections from each animal were stained by immunofluorescence for CD2 and CD8 as described above. Fluorescent cells in each region as illustrated in Supplementary Figure S1 were counted by an investigator blinded to the sample identity, using an Axio Imager Z1 fluorescence microscope (Zeiss, Oberkochen, Germany). Sections were viewed with the 20× objective and the counting was made “on view” directly at the microscope.

Pictures of three Iba1-labelled lumbar sections for each SNI and sham operated animals were taken with the 20× objective, keeping the same fluorescence intensity and exposure for every picture. For Iba1 signal quantification, the contrast of images was enhanced using GNU Image Manipulation Program software (www.gimp.org). The labelled area was quantified and expressed as a percentage of total area using ImageJ software.

### 4.3. RT-qPCR and RT-qPCR Array

For RNA samples, rats were terminally anesthetized by a 50 mg i.p. injection of pentobarbital and transcardially perfused with NaCl 0.9%. The L4 and L5 ipsilateral dorsal horns of the spinal cord were dissected and rapidly frozen. The tissues were homogenized and total RNA was extracted with RNeasy Plus Mini Kit (Qiagen^®^, Hombrechtikon, Switzerland). The obtained RNA, was purified by the Minelute Reaction Cleanup Kit (Qiagen^®^), then analyzed by the RNA 6000 Nano Assay (Agilent^®^,Basel, Switzerland), and reverse transcribed into cDNA with Omniscript RT Kit (Qiagen^®^), if RNA integrity number (RIN) was over 7.5. The RT-qPCR amplification was performed with ROX SYBR Green on an ABI 7900 HT (Bio-Rad^®^, Reinach, Switzerland).

### 4.4. Statistics

All data are represented as mean ± 95% CI. For the RT-qPCR of the initial time course, the non-overlap of 95% CI method was used [13]. No other statistical test was performed on these data due to the small sample size (3 animals/group). Two-way ANOVA was used for Iba1 immunohistochemistry, BrdU time course, and T-lymphocyte counts. A *p*-value <0.05 was considered significant. The RT2 profiler PCR array data analysis version 3.4 from SABiosciences (Qiagen^®^) was used for the array (6 animals/group for each SNI time point and 4/group for sham D2 and D4). It does not apply any correction for multiple testing and a *p*-value of 0.05 is proposed as significant. We however also provide the exact *p*-values of the array data if the reader prefers to apply more conservative statistics (Table S1). For the four time points time course, a *p*-value <0.0125 could be taken as significant (Bonferroni correction). All RT-qPCR data were analyzed with the delta *C*_t_ method.

## Figures and Tables

**Table 1:** Primary antibodies used for immunofluorescence.

Antibody	Source	Target	Concentration
Mouse anti-CD2	Serotec, UK	T-cells	1:250
Mouse anti-CD8	Abcam, USA	CD8+ T-cells	1:250
Rabbit anti-Iba1	Wako, USA	Activated microglia	1:2000
Mouse anti-GFAP	Millipore, USA	Activated astrocytes	1:1500
Rat anti-BrdU	Abcam, USA	Proliferating cells	1:500

**Table 2:** Secondary antibodies used for immunofluorescence.

Antibody	Source	Concentration
Alexa 488-labelled goat anti-mouse	Molecular Probes, UK	1:500
Alexa 488-labelled donkey anti-rabbit	Molecular Probes, UK	1:500
Cy3-labelled donkey anti-mouse	Jackson, USA	1:500
Cy3-labelled donkey anti-rat	Jackson, USA	1:500

## Supplementary Materials

Supplementary materials can be found at http://www.mdpi.com/1422-0067/17/3/352/s1.

## Abbreviations

BrdU	Bromodeoxyuridine
CI	Confidence interval
CCI	Chronic constriction injury
CHUV	Centre Hospitalier Universitaire Vaudois
CNS	Central nervous system
LPS	Lipopolysaccharides
MAC	Membrane attack complex
MDPI	Multidisciplinary Digital Publishing Institute
mRNA	Messenger ribonucleic acid
PBS	Phosphate buffer saline
PSNL	Partial sciatic nerve ligation
RT-qPCR	Real-time quantitative polymerase chain reaction
SNI	Spared nerve injury
SCI	Spinal cord injury
TLR	Toll-like receptor
