# Supplementary material for: Spinal Cord T-Cell Infiltration in the Rat Spared Nerve Injury Model: A Time Course Study
Source: Int J Mol Sci. 2016 Mar 9;17(3):352. doi: 10.3390/ijms17030352 (PMC4813213; doi:10.3390/ijms17030352)
Supplement: Supplementary file 1 [file ijms-17-00352-s001.pdf]

Supplementary Materials: Spinal Cord T-Cell Infiltration in the Rat Spared Nerve Injury Model: A Time Course Study

Christophe Gattlen, Christine B. Clarke, Nicolas Piller, Guylène Kirschmann, Marie Pertin, Isabelle Decosterd, Romain-Daniel Gosselin and Marc R. Suter

Table S1. Genes from qPCR array shown in Figures 4, 5A and S2. Exact *p*-values for genes from qPCR array shown in Figures 4, 5A and S2, *p*-value (Student's *t*-test, compared to control group), in red $p < 0.05$.

Figure 4						
**Symbol**	**SNI D2**	**SNI D4**	**SNI D10**	**SNI D 21**	**Sham D2**	**Sham D4**
Il6	0.2433	0.0039	0.0036	0.0010	0.5957	0.3724
Il18	0.0016	0.0198	0.0025	0.0071	0.4632	0.4784
Tnf	0.0170	0.4089	0.8747	0.7619	0.3022	0.1002
Ccl1	0.3796	0.1863	0.2031	0.0306	0.2854	0.2573
Ccl22	0.6404	0.2077	0.3666	0.0027	0.6753	0.3053
Il10	0.3397	0.2279	0.2417	0.0085	0.5318	0.0525
Tlr1	0.0019	0.0009	0.0000	0.0000	0.0311	0.0580
Tlr2	0.4178	0.0044	0.0003	0.0004	0.9363	0.2020
Tlr6	0.2838	0.0454	0.0180	0.0154	0.9007	0.4232
Tlr7	0.0017	0.0007	0.0000	0.0000	0.4632	0.3094
Ly96	0.1382	0.0430	0.0032	0.0012	0.9342	0.1205
Myd88	0.1895	0.0174	0.0018	0.0099	0.4420	0.3298
Nfkb1	0.9153	0.3075	0.0735	0.0345	0.5872	0.3078
C3	0.8378	0.0017	0.0004	0.0001	0.5649	0.2429
C3ar1	0.0448	0.0612	0.0187	0.0352	0.5166	0.3245
C4b	0.1637	0.0040	0.0052	0.0001	0.9588	0.3336
Cd68	0.2881	0.0029	0.0000	0.0031	0.1039	0.0952
Aif1 (Iba1)	0.0230	0.0041	0.0003	0.0013	0.0400	0.2237
CX3CR1	0.0492	0.0008	0.0003	0.0000	0.8152	0.3263
**Figure 5**						
Ccl12	0.0129	0.3837	0.1046	0.1302	0.1134	0.0021
Ccl22	0.6404	0.2077	0.3666	0.0027	0.6753	0.3053
Cxcl11	0.0109	0.3697	0.9909	0.8491	0.7270	0.3950
**Figure S2**						
Ccl12	0.0129	0.3837	0.1046	0.1302	0.1134	0.0021
Ccl4	0.0347	0.9222	0.0782	0.1635	0.6786	0.0619
Cd40	0.0000	0.0517	0.0003	0.0008	0.9316	0.2945
Cebpb	0.0017	0.1056	0.1136	0.4229	0.3091	0.3591
Csf1	0.5155	0.2408	0.0925	0.0109	0.5738	0.2444
Fos	0.2804	0.0502	0.0956	0.0008	0.9424	0.2281
Il10	0.3397	0.2279	0.2417	0.0085	0.5318	0.0525
Il18	0.0016	0.0198	0.0025	0.0071	0.4632	0.4784
Il18rap	0.0037	0.0650	0.0042	0.0000	0.5257	0.9746
Il1b	0.6978	0.6485	0.1329	0.1768	0.8802	0.9859
Il1r1	0.1614	0.1969	0.1208	0.0024	0.8188	0.2289

Table S1. *Cont.*

**Figure S2**						
Il23r	0.7922	0.0153	0.0123	0.0162	0.6401	0.0745
Il6	0.2433	0.0039	0.0036	0.0010	0.5957	0.3724
Il6r	0.2254	0.0304	0.0070	0.0363	0.1606	0.8766
Itgb2	0.0003	0.0160	0.0019	0.0028	0.4380	0.5729
Ripk2	0.1257	0.2770	0.0265	0.0060	0.1976	0.6640
Tlr1	0.0019	0.0009	0.0000	0.0000	0.0311	0.0580
Tlr2	0.4178	0.0044	0.0003	0.0004	0.9363	0.2020
Tlr3	0.2597	0.1794	0.5503	0.5637	0.5871	0.7104
Tlr4	0.9409	0.1764	0.0151	0.0023	0.1956	0.2297
Tlr5	0.7136	0.0198	0.7916	0.2317	0.1417	0.1894
Tlr6	0.2838	0.0454	0.0180	0.0154	0.9007	0.4232
Tlr7	0.0017	0.0007	0.0000	0.0000	0.4632	0.3094
Tnfsf14	0.0285	0.1414	0.0316	0.0366	0.2888	0.4997

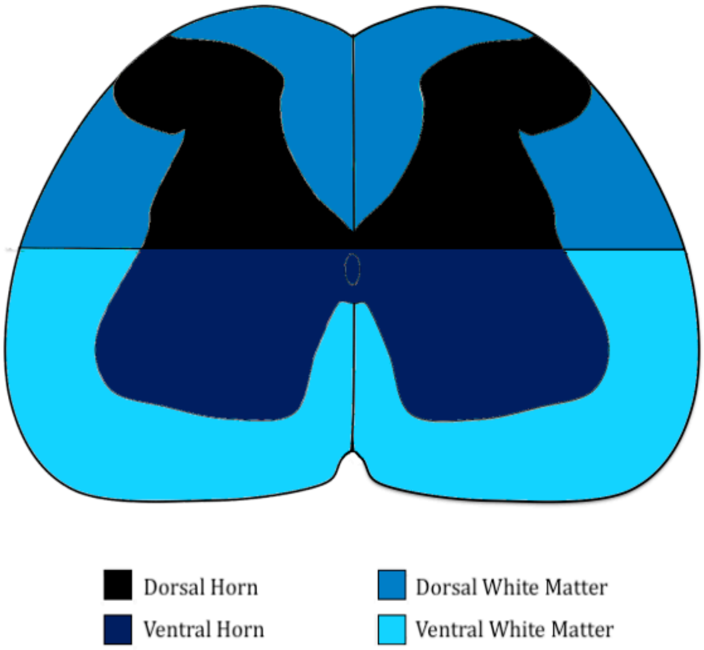

Figure S1. Definition of regions for cell counting.

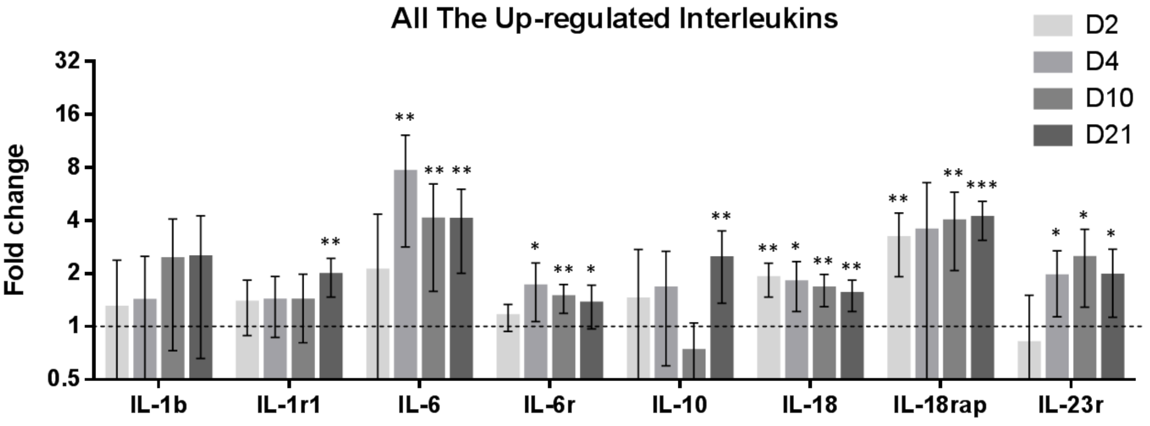

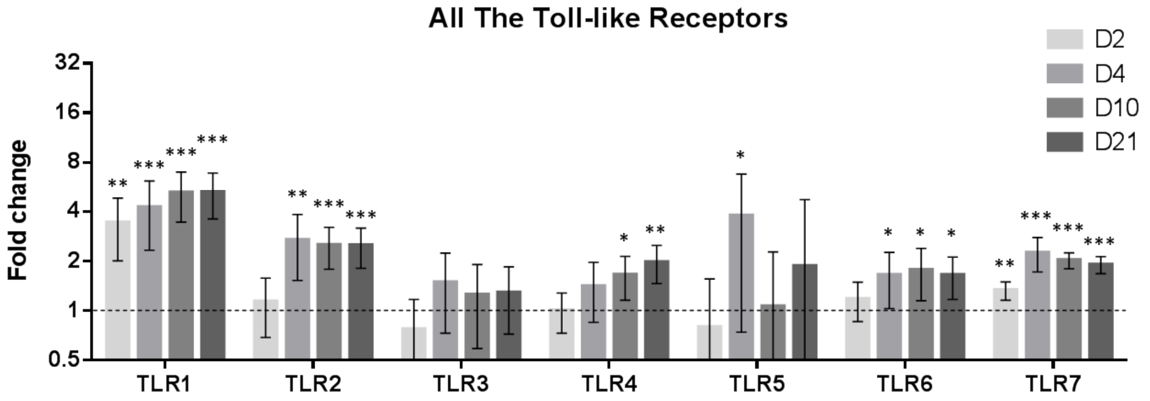

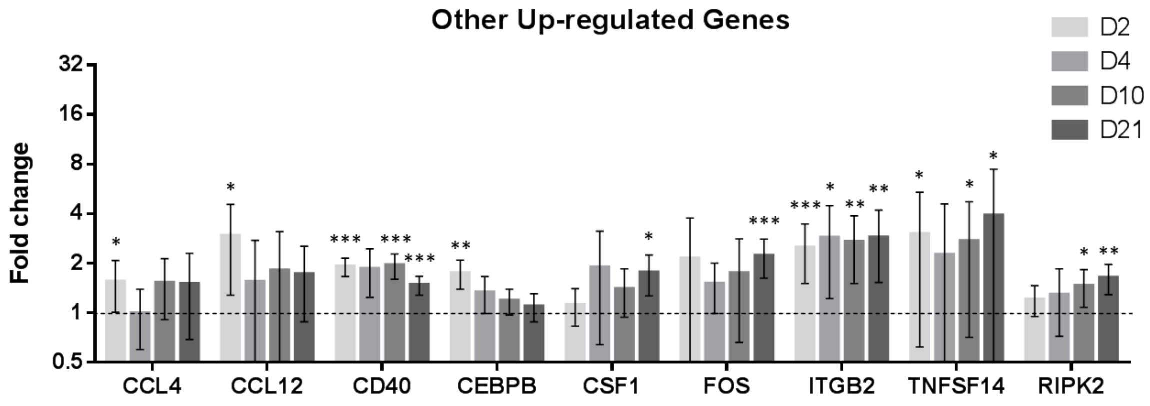

Figure S2. Gene regulation in the spinal cord following SNI- other upregulated genes. RT-qPCR array with fold change of gene expression in adult male rats at D2, D4, D10 and D21 after SNI compared to naive animals. Values are expressed as mean ± 95% CI. Dotted line at 1 fold regulation (naive animals). * $p < 0.05$, ** $p < 0.01$, *** $p < 0.001$. Student's *t*-test: Naive (not shown) *vs*. SNI. $N = 6$ for each time point (D2, D4, D10 and D21).

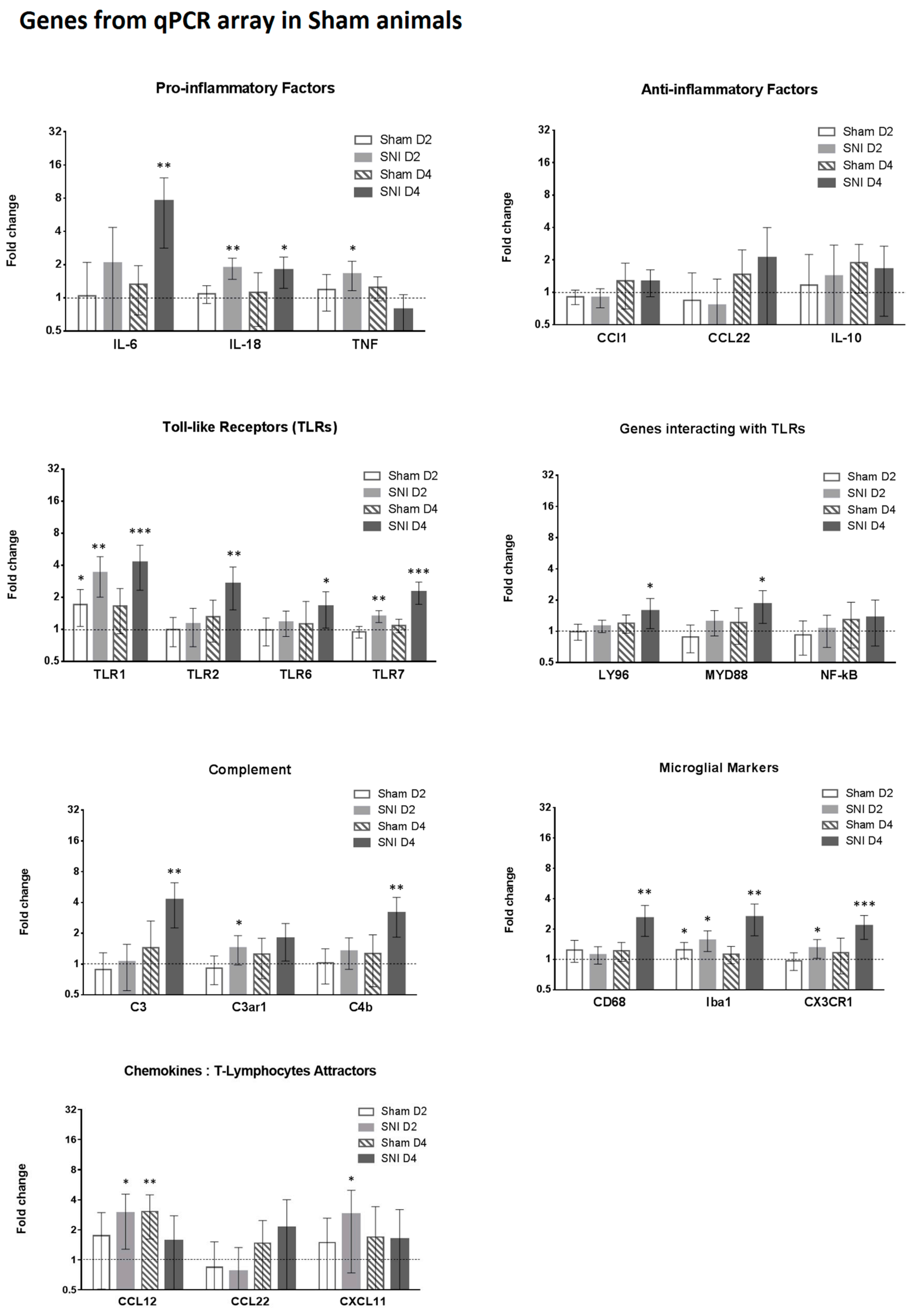

Figure S3. Genes from qPCR array in sham animals. RT-qPCR array with fold change of gene expression of adult male rats at D2 and D4 after sham and SNI compared to naive animals (not shown). Values are expressed as mean ± 95% CI. Dotted line at one fold regulation (naive animals). * $p < 0.05$, ** $p < 0.01$, *** $p < 0.001$. Student's *t*-Test naive versus sham or SNI, N=6 for SNI D2 and D4. *N*= 4 for sham D2 and D4.

Supplementary Materials and Methods: Western Blotting

One week after SNI, rats were terminally anesthetized by an i.p. injection of pentobarbital and then sacrificed by decapitation. Spinal cords were dissected to collect the L4–L5 lumbar enlargement. Following extraction, the protein concentration for each sample was measured by a Bradford protein assay. After migration and transfer onto Immuno-Blot PVDF membranes (Bio-Rad, Germany), blocking was performed in 5% non-fat milk for 5 min at room temperature. Incubation with 1:200 Rabbit anti-CD2 (Santa-Cruz, CL, USA) was done in 4 °C milk overnight.

For detection, membranes were incubated for 30 min at room temperature in horseradish peroxidase-conjugated secondary antibody (1:10,000 goat anti-rabbit/HRP, Dako, Denmark). Washings were made 5 times between each incubation in tween 0.1% 2× PBS. Signals were revealed using enhanced chemiluminescence (ECL) reagent (West Dura, Pierce, Rockford, IL, USA). For imaging, we used a luminescent image analyzer (LAS-4000, Fujifilm, Japan).

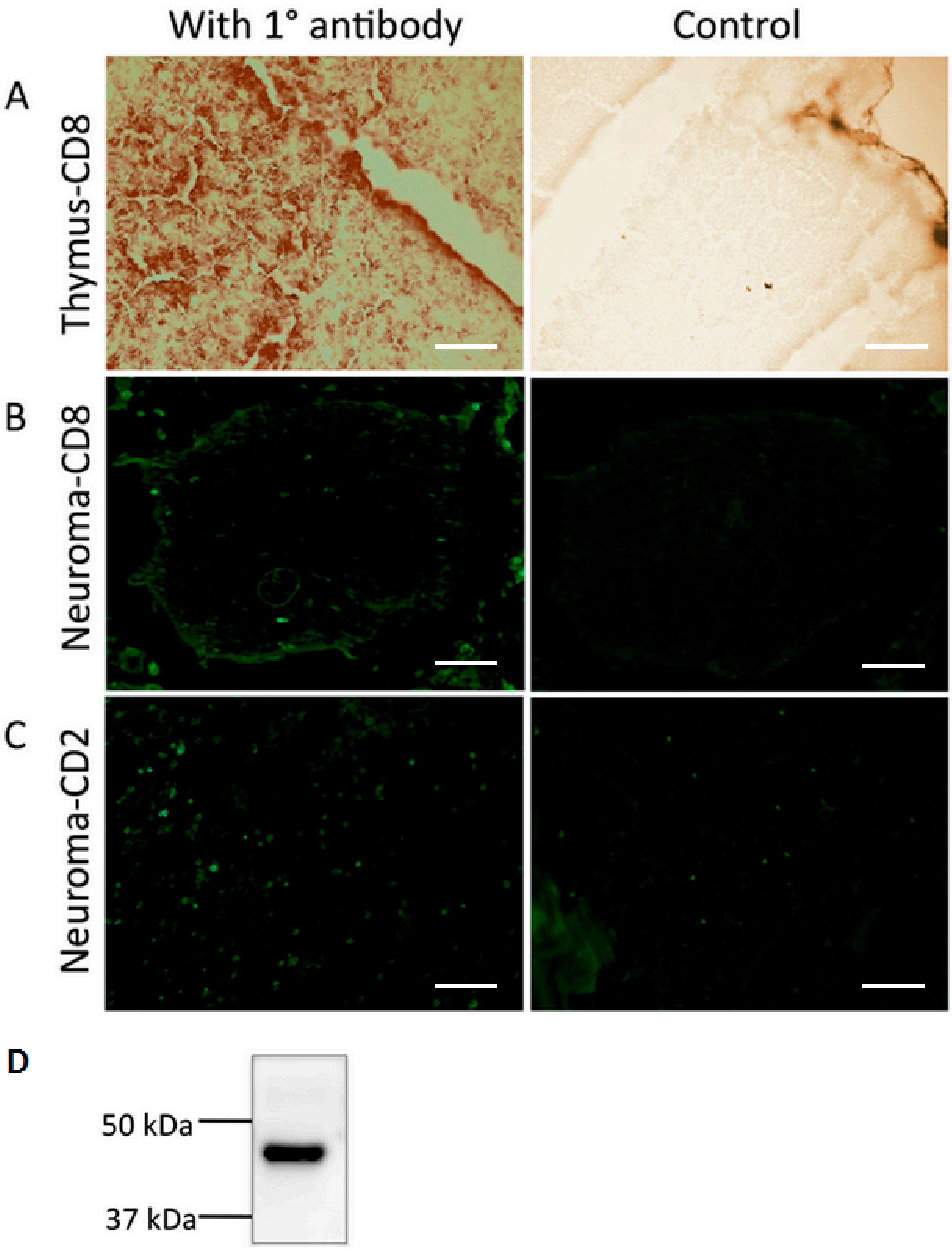

Figure S4. Antibody sensitivity tests. (**A**) Immunochemistry on rat thymus with (**left**), or without (**right**) primary anti-CD8 antibody; (**B**,**C**) Immunofluorescence on neuroma two days after SNI with green-labeled CD2 or CD8 T-cell marker (**left**), and control without primary antibody (**right**). Small cells consistent with a lymphocytic morphology are targeted; (**D**) Western blot analysis seven days after SNI of thymus protein extract with anti-CD2 antibody, showing a single band around 45 kDa. Scale bar represents 100 μm.

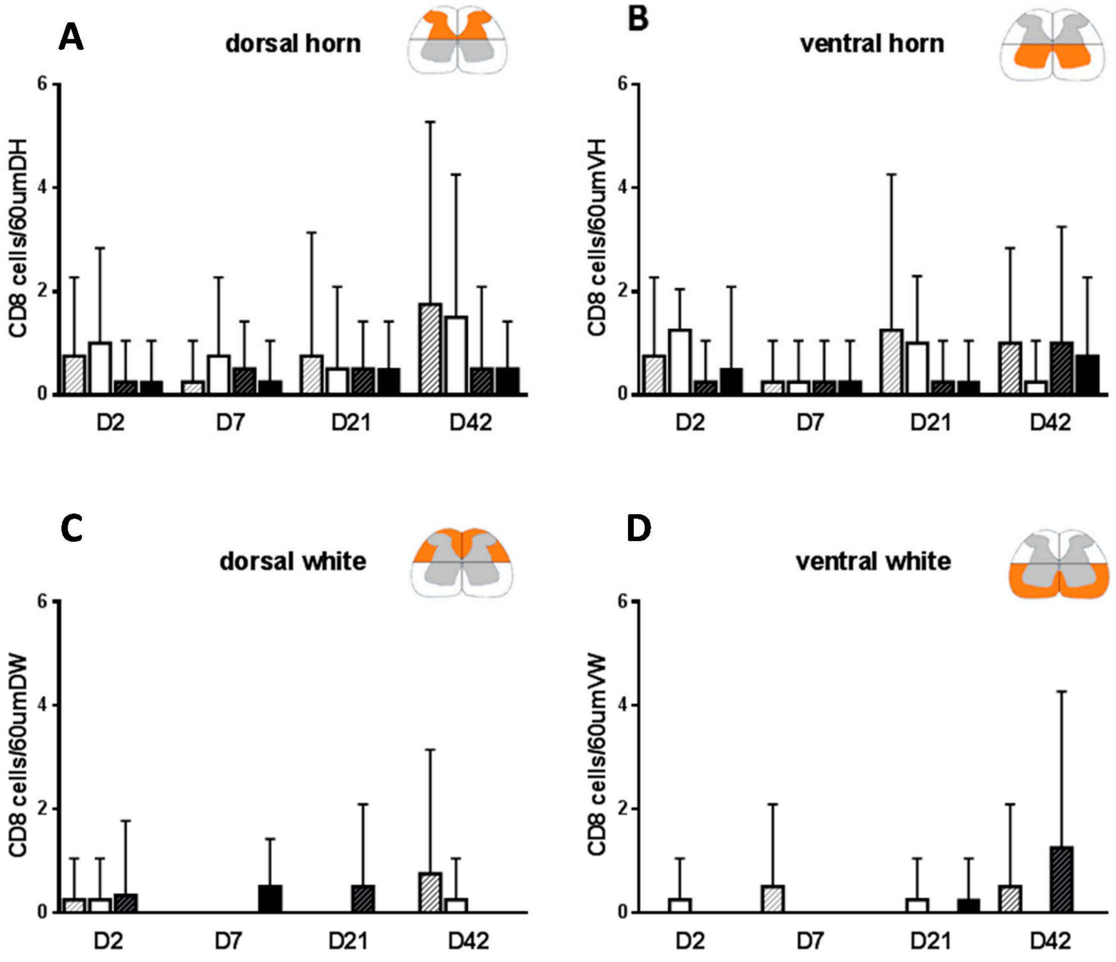

Figure S5. Time course of CD8-positive T-cell distribution in the lumbar spinal cord. (**A**–**D**): Bar histograms showing the number of CD8+ cells detected in sham (white) or SNI (black) animals at days 2, 7, 21 and 42, ipsilateral (hatched) or contralateral (full) to injury. Four regions of the spinal cords were analyzed: (**A**) the dorsal horn (DH); (**B**) the ventral horn (VH); (**C**) the dorsal white matter (DW) and (**D**) the ventral white matter (VW). In several cases, no cells were detected (where no bar is shown). N = 4/groups. Values are expressed as mean ± 95% CI of cells counted in two 30µm lumbar sections per animal. 2-way ANOVA with Sidak correction. No significant difference was found between sham and SNI.

Supplementary Materials and Methods: IB4 Immunostaining

To assess the part of the spinal cord with afferent injured nerves, a staining with isolectin (Griffonia simplicifolia) B4 FITC conjugated has been performed on D2, D7, D21 and D42 SNI and sham slices. After blocking the slice for half an hour, the IB4 was incubated for 2 hours, the slides were then washed in PBS and mounted in mowiol. One of each of the 5 slices in mean was stained, therefore, scanning the area of interest.

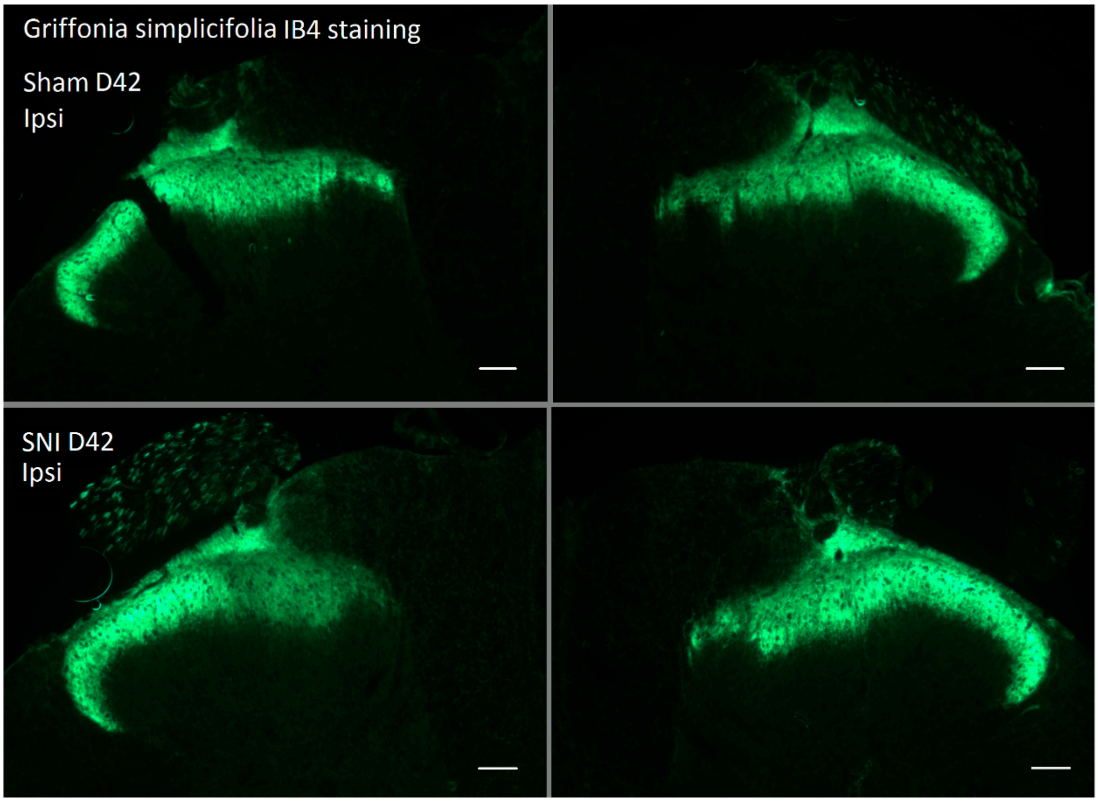

Figure S6. IB4 staining on D42 Sham and SNI rat dorsal horn. The Griffonia (Bandeiraea) simplicifolia (isolectin B4) staining which shows the typical loss of staining in the superficial laminae of the dorsal horn after peripheral nerve injury in the ipsilateral side (**bottom left**).Scale bar represents 50 µm.
